# A comprehensive bibliometric analysis of global research on the role of acrolein in Alzheimer’s disease pathogenesis: involvement of amyloid-beta

**DOI:** 10.3389/fnagi.2024.1378260

**Published:** 2024-05-09

**Authors:** Amadou Wurry Jallow, Doan Phuong Quy Nguyen, Monika Renuka Sanotra, Chun-Hsien Hsu, Yi-Fang Lin, Yung-Feng Lin

**Affiliations:** ^1^Ph.D. Program in Medical Biotechnology, College of Medical Science and Technology, Taipei Medical University, New Taipei City, Taiwan; ^2^Institute of Biomedicine, Hue University of Medicine and Pharmacy, Hue University, Hue, Vietnam; ^3^Department of Medical Genetics, Hue University of Medicine and Pharmacy, Hue University, Hue, Vietnam; ^4^Department of Cardiology, Taipei Veterans General Hospital, Taipei, Taiwan; ^5^Department of Family Medicine, Heping Fuyou Branch, Taipei City Hospital, Taipei, Taiwan; ^6^School of Medicine, College of Medicine, Fu Jen Catholic University, New Taipei City, Taiwan; ^7^Department of Exercise and Health Sciences, University of Taipei, Taipei, Taiwan; ^8^Department of Laboratory Medicine, Taipei Medical University Shuang Ho Hospital, New Taipei City, Taiwan; ^9^School of Medical Laboratory Science and Biotechnology, College of Medical Science and Technology, Taipei Medical University, New Taipei City, Taiwan; ^10^Department of Laboratory Medicine, Taipei Medical University Hospital, Taipei, Taiwan

**Keywords:** Alzheimer’s disease, acrolein, oxidative stress, bibliometrics, amyloid-beta, Web of Science

## Abstract

**Background:**

Alzheimer’s disease (AD) is a progressive neurodegenerative disorder characterized by cognitive and behavioral decline. Acrolein, an environmental pollutant and endogenous compound, is implicated in AD development. This research employs bibliometric analysis to assess current trends and key areas concerning acrolein-AD interaction.

**Methods:**

The Web of Science was used to extensively review literature on acrolein and AD. Relevant data were systematically gathered and analyzed using VOSviewer, CiteSpace, and an online bibliometric tool.

**Results:**

We identified 120 English publications in this specialized field across 19 journals. The Journal of Alzheimer’s Disease was the most prominent. The primary contributors, both in terms of scientific output and influence, were the USA, the University of Kentucky, and Ramassamy C, representing countries/regions, institutions, and authors, respectively. In this field, the primary focus was on thoroughly studying acrolein, its roles, and its mechanisms in AD utilizing both *in vivo* and *in vitro* approaches. A significant portion of the research was based on proteomics, revealing complex molecular processes. The main focuses in the field were “oxidative stress,” “lipid peroxidation,” “amyloid-beta,” and “cognitive impairment.” Anticipated future research trajectories focus on the involvement of the internalization pathway, covering key areas such as synaptic dysfunction, metabolism, mechanisms, associations, neuroinflammation, inhibitors, tau phosphorylation, acrolein toxicity, brain infarction, antioxidants, chemistry, drug delivery, and dementia. Our analysis also supported our previous hypothesis that acrolein can interact with amyloid-beta to form a protein adduct leading to AD-like pathology and altering natural immune responses.

**Conclusion:**

This study provides a broad and all-encompassing view of the topic, offering valuable insights and guidance to fellow researchers. These emerging directions underscore the continuous exploration of the complexities associated with AD. The analyses and findings aim to enhance our understanding of the intricate relationship between acrolein and AD for future research.

## Introduction

Alzheimer’s disease (AD) is a complex and progressive neurodegenerative disorder that primarily affects the brain, leading to cognitive decline and behavioral changes. It is the most common cause of dementia, a general term for a decline in cognitive ability severe enough to interfere with daily life (Alzheimer’s Association, 2013; Morató et al., 2022). The hallmark features of AD include the accumulation of abnormal proteins aggregates in the brain, namely amyloid plaques and tau tangles. These aggregates interfere with the normal functioning of neurons and disrupt communication between brain cells. This results in the gradual loss of memory, thinking skills, and eventually the ability to carry out basic tasks (Querfurth and LaFerla, 2010; Selkoe and Hardy, 2016). The exact cause of AD is not fully understood, but age, genetic, lifestyle and environmental factors are known to be major risk factors. While there is currently no cure for AD, there are treatments and interventions available that can help manage some of the symptoms and improve the quality of life for both individuals with the disease and their caregivers.

Acrolein is an alpha, beta-unsaturated aldehyde, considered both an environmental pollutant and an endogenous substance. It is highly reactive, volatile, and toxic, characterized by a pungent odor. Exogenously, it can be produced during the incomplete combustion of organic materials, including cigarette smoke, automobile exhaust, and emissions from industrial processes. Additionally, acrolein can be formed during certain cooking processes, such as frying or grilling, especially when oils and fats are heated to high temperatures (Stevens and Maier, 2008; Kassem et al., 2018). Within the body, it can be produced as the end product of lipid peroxidation, threonine degradation, anticancer drug metabolism, and polyamine catabolism (Chang et al., 2022).

Alzheimer’s disease is characterized by a complex interplay of pathological factors. Nevertheless, a prevailing hypothesis suggests that the progressive neuronal degeneration observed in AD may be attributed to the accumulation of extracellular amyloid plaques primarily composed of amyloid-ß (Aß). These plaques are believed to originate from the proteolytic cleavage of amyloid precursor protein (APP) mediated by ß- and γ-secretases (Wolfe, 2003; Sanotra et al., 2023). There is evidence suggesting that acrolein plays a significant role in the pathogenesis of various neurodegenerative diseases, including AD (Chang et al., 2022). In AD, acrolein levels are notably elevated in the hippocampus and temporal cortex regions of the brain. This increase in acrolein contributes to AD by elevating oxidative stress, damaging neurons, and affecting various biological pathways. The detrimental effects of acrolein encompass DNA damage, disruption of mitochondrial function, initiation of endoplasmic reticulum stress, protein adduction, promotion of inflammation, impairment of cell membranes, formation of reactive oxygen species (ROS), and tau phosphorylation, thereby influencing the pathophysiology of AD (Dang et al., 2010; Moghe et al., 2015). Notably, a research study has highlighted the development of a straightforward sporadic AD animal model through acrolein administration. This model exhibited classic AD pathologies, including increased levels of amyloid-beta (Aβ) and tau phosphorylation, proliferation of astrocytes and microglia, reduced synaptic proteins, and cognitive impairments (Huang et al., 2013; Chen et al., 2022). The model proved to be useful for studying the mechanisms underlying the onset of AD and the potential development of anti-AD drugs. Moreover, research has investigated the alterations in acrolein metabolism in AD. It is suggested that deregulated acrolein metabolism may be correlated with neuronal damage in AD patients, providing potential insights into the disease progression and early diagnosis of AD (Sexton et al., 2021; Sanotra et al., 2023). Acrolein has been implicated in the development of AD following traumatic brain injury (TBI) or as a secondary factor in TBI-related AD. Recent research has shown that acrolein, acting as a diffusive factor in secondary injury, plays a crucial and self-sufficient role in promoting inflammation (TNF-α) and Aβ42 aggregation, key contributors to AD pathology (Rogers et al., 2023). However, the exact interaction between acrolein and AD remains unclear.

Bibliometric analysis serves as a valuable tool for assessing the influence of publications and research collectives within their respective fields (Ho and Kahn, 2014). Moreover, it offers an effective means of quantifying the caliber of published output for entities such as countries, institutions, and authors (Zhang and Wang, 2012). Presently, the utilization of the Science Citation Index Expanded or Social Science Citation Index has become indispensable in gauging the research achievements of countries, institutions, and authors across multiple dimensions (Jallow et al., 2021). Recent times have witnessed the execution of bibliometric analyses within the Web of Science (WoS) categories, including psychology (Berta et al., 2022), neurosciences (Wilson et al., 2021; Fei et al., 2022), cardiology (Wang et al., 2022), and cancer (Fresno-Alba et al., 2023; Xu et al., 2023). These analyses have played a crucial role in evaluating research achievements and productivity across countries, institutions, and authors. In a similar vein, this study will center its focus on dissecting research papers concerning the role of acrolein in the genesis of AD.

Keyword search plays a pivotal role in bibliometric analysis for multiple reasons. It enables researchers to focus their investigation on specific topics, thus enhancing the accuracy and relevance of their findings. Keywords serve as gateways to the vast realm of scholarly literature, allowing researchers to efficiently identify and retrieve relevant publications. By using the right keywords, researchers can reduce noise, pinpoint key articles, and gain valuable insights into research trends, impact, and collaborations (Ellegaard and Wallin, 2015). Using precise keywords and targeted databases to investigate the correlation between acrolein and the pathogenesis of AD can provide valuable insights into the condition, enabling the identification of potential protective strategies.

This study covers research papers published up to the current date, which is 31 July 2023. The primary aim is to investigate the impact of acrolein exposure on the pathogenesis of AD. The results of this research have the potential to enhance the understanding of the role of acrolein in the development of AD, thereby promoting the creation of novel therapeutic interventions.

## Materials and methods

### Data source

The bibliometric information for research reports was obtained from the Science Citation Index (SCI) Expanded database within the Web of Science Core Collection of Clarivate Analytics. This database is a rich collection of citation indexes that represents the connections between scholarly research articles from various fields, including sciences, social sciences, and arts and humanities (Poly et al., 2023).

### Search strategy

**FIGURE 1:**
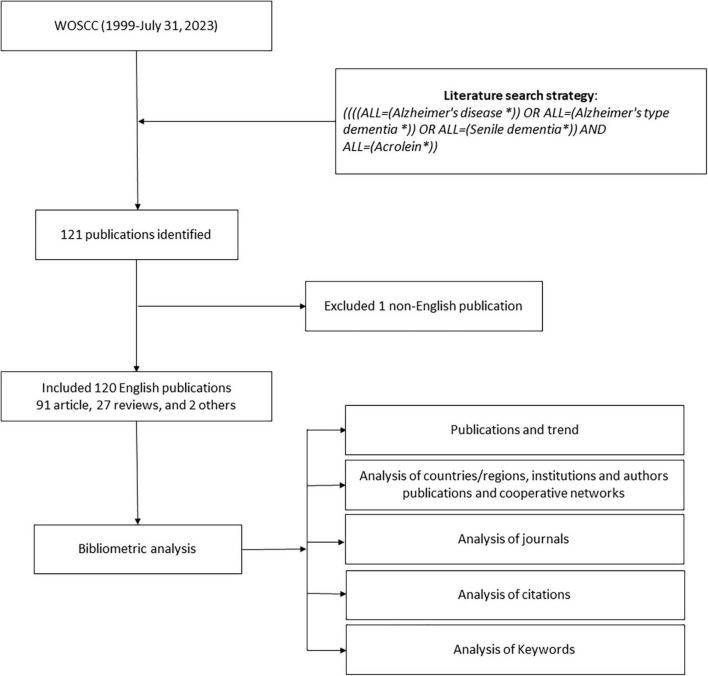
Flow chart of literature search and bibliometric analysis.

In this study, we utilized the search keywords “Acrolein*” and “Alzheimer’s *” OR “Alzheimer’s type dementia *” OR “Senile dementia*” to conduct a topic search across various fields, including title, abstract, author keywords, and KeyWords Plus. The objective of this search was to identify research papers related to the pathogenesis of AD and the role of acrolein in this context. No time limitation was set for the search, thereby encompassing a wide range of relevant literature. To ensure the consistency and reliability of the search results, two different researchers independently performed the search process. This approach aimed to minimize any potential biases or discrepancies in the selection of research papers. The researchers completed the search process on 31 July 2023. After identifying the relevant research papers, they were exported to Excel for further analysis and investigation. A complete representation of literature search and bibliometric analysis process is shown in Figure 1.

Our retrieval formula:


$$\left((((ALL\text{ = (}Alzheimer'sdisease^{*}\text{)}\right)$$

$$OR\;ALL\text{ = }(Alzheimer'stype\;dementia^{*}\text{)})$$

$$OR\;ALL\text{ = }(Senile\;dementia^{*}\text{)})\;ANDALL\text{ = (}Acrolein^{*}\text{))}$$

### Data collection and bibliometric index statistics

In the research process, the relevant data from all the searched documents were exported, and various bibliometric indicators were identified and computed using Microsoft Excel. These indicators encompassed crucial aspects of the publications, including the following: annual number of publications, citation frequency, average citation frequency, journal name, journal impact factor, publication country/region, publication institution, and author. By employing Excel, the researchers were able to efficiently organize and analyze the bibliometric data. This analysis provides valuable insights into research performance, citation patterns, and the impact of scholarly works across various fields of study.

### Visualization analysis

In our study, we made use of three powerful bibliometric analytic tools to conduct a comprehensive analysis of scholarly data. These tools were VOSviewer (Version 1.6.16), CiteSpace (version 6.2.R6), and an online bibliometric tool available at https://bibliometric.com/. VOSviewer, which is a versatile software tool, played a key role in mapping institutions’ cooperation, authors’ cooperation, co-authorship, citation, co-citation, and bibliographic coupling (van Eck and Waltman, 2010). With VOSviewer, we were able to visualize and explore complex networks of collaborations and relationships within the scholarly landscape, gaining valuable insights into the interconnections between authors, institutions, and publications. For a deeper understanding of emerging trends and research hotspots within our field of study, we used VOSviewer for keyword co-occurrence analysis and the CiteSpace software tool for clustering analysis and keyword burst detection. Additionally, to map country/region and institution cooperation, we employed an online bibliometric tool, which offered unique features complementing the analyses conducted with VOSviewer.

### Analysis of KeyWords Plus

KeyWords Plus is a specialized application used for citation indexing, which involves extracting relevant terms from the titles of publications cited by authors in the ISI (now Clarivate Analytics) database. The primary goal of KeyWords Plus is to enhance the citation indexing process, improving its accuracy and efficiency, as mentioned by Garfield in his works (Bose and Sarma, 1975; Makar et al., 1975). Analysis of keyword in article title also serve as useful tool for identification of research hotspots and frontiers in a certain filed. In the present study, we utilized the VOSviewer analytic tool to extract keywords from the 120 articles. To identify the most prominent keywords, we strictly adjust selection threshold a minimum of 5 occurrences. This process resulted in the retrieval of the most prominent keywords related to acrolein and AD.

CiteSpace software tool is another powerful tool for clustering research hot spots, identifying changes of the research direction by timeline, and predicting the research frontiers in the field. With the use of CiteSpace software tool, each cluster was ranked based on the elements and assigned a research category associated with its content. The names of the research categories for each cluster were determined according to log-likelihood ratio and mutual information. The clusters were arranged in ascending order of ID number, with smaller ID numbers indicating larger clusters and vice versa. Additionally, the software calculated the silhouette value to assess the homogeneity of each cluster. This metric evaluates how similar a keyword is to its own cluster compared to other clusters. A higher silhouette value indicates greater consistency and confidence in the keywords within the cluster.

## Results

### Publication output, document type, and language

**FIGURE 2:**
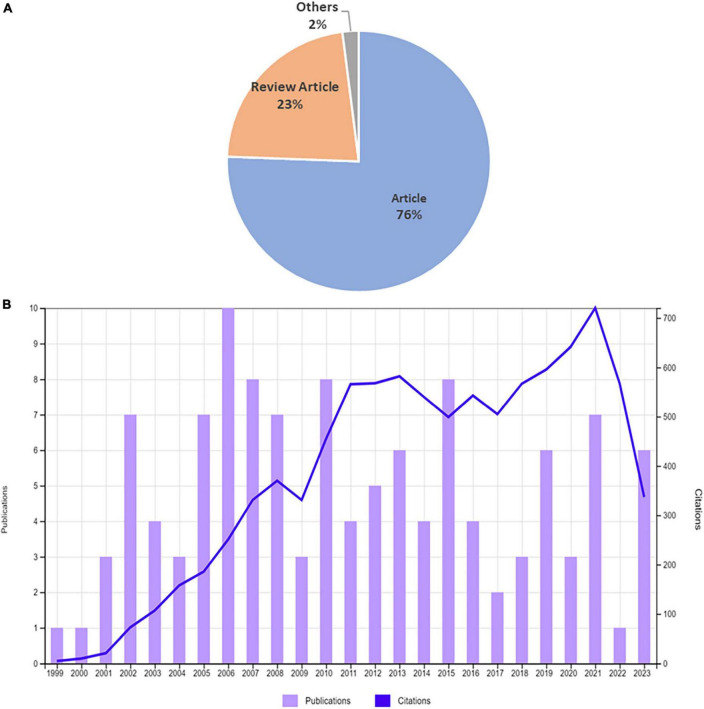
Publication distribution. Document type **(A)** and total articles per annum **(B)** in the field of acrolein and Alzheimer’s disease research.

The study covered research papers published between the years 1999 and July 2023. We found a total of 121 publications related to acrolein and AD in SCI-Expanded of Web of Science. These publications were indexed within six document types in the WoS. Among these document types, the category “Article” accounted for 91 publications, representing 76% of the total publications. Additionally, there were 27 publications categorized as “Review,” constituting 23% of the 120 publications and 2 publications for “Others,” representing 2% (Figure 2A). All the documents were originally written in English except one which is rewritten in Chinese.

Figure 2B illustrates the annual count of research publications related to acrolein and AD. The data indicates a noticeable decline in the number of articles published in recent years compared to the preceding decade when these topics garnered significant attention.

### Publication of countries/regions

**FIGURE 3:**
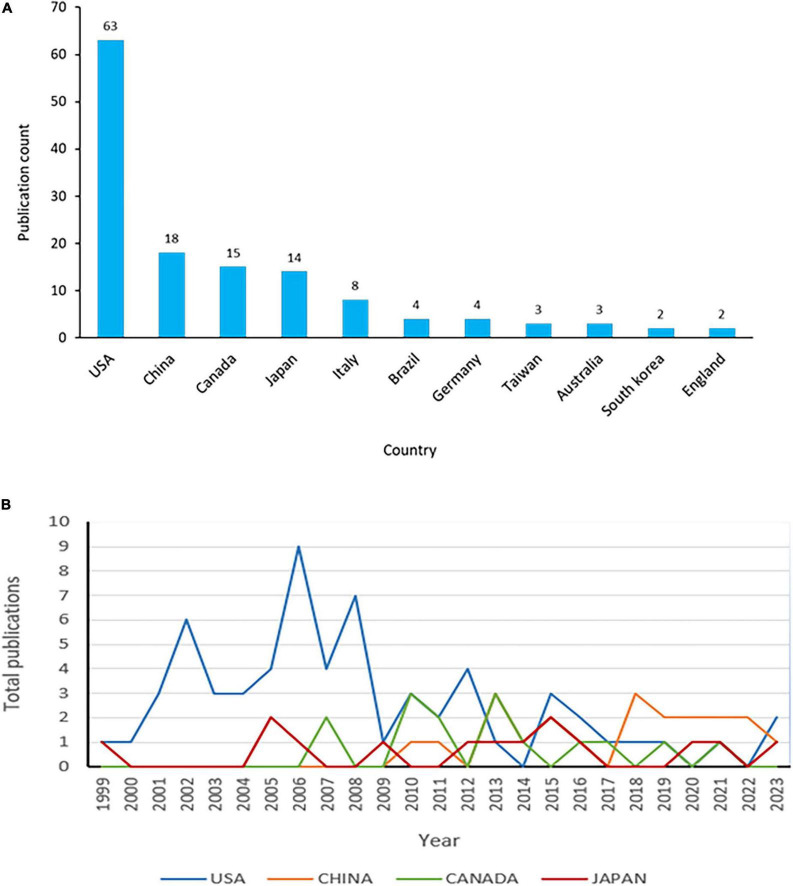
Top 10 countries with highest publications in acrolein and Alzheimer’s disease research field. **(A)** Publication count of each country. **(B)** Annual publication trend of the top four countries.

**FIGURE 4:**
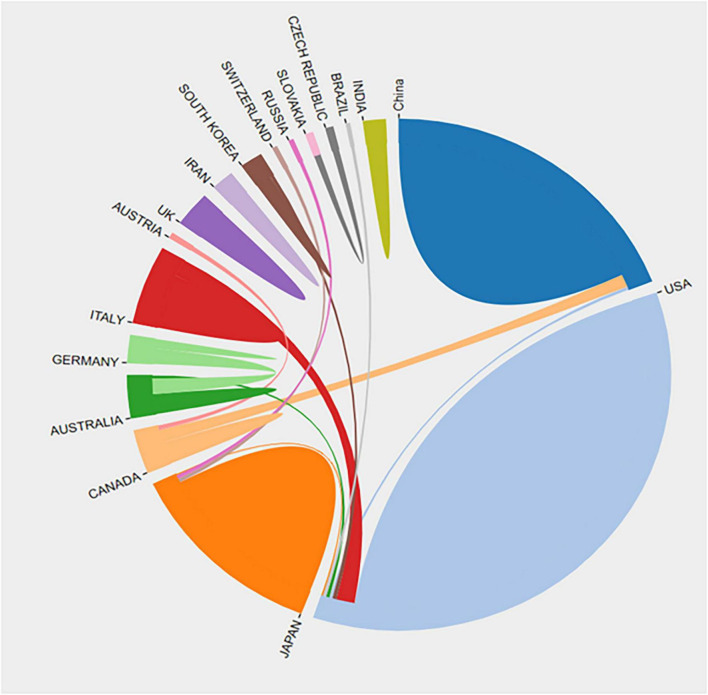
Inter-country/region publication in the acrolein and Alzheimer’s disease research field. The links between countries are represented by colored lines. More linked lines indicate greater collaboration.

There was a total of 22 countries that contributed to the publications on acrolein and AD. Among these countries, the majority of the articles were published by the USA, accounting for 52.5% (*n* = 63) of the total publications. In addition to the USA, other countries like China, Canada, and Japan also made noteworthy contributions, accounting for 15% (*n* = 18), 12.5% (*n* = 15), and 11.7% (*n* = 14) of the publications, respectively (Figure 3A). In Figure 3B, the annual publication trend of the four countries with the highest number of publications between 1999 and July 2023 is displayed. The data reveals that the USA experienced a substantial increase in publications during the last two decades but has since been declining in recent years, whereas China and Japan have consistently achieved significant publication outputs. Moreover, despite the recent decline in the USA’s publications, they received the highest number of international collaborations in the field. Japan ranked second in terms of international collaborations, followed by China and Canada. Interestingly, countries like the UK, India, and Iran had no collaborations at all (Figure 4).

This indicates that research on acrolein and AD is a global effort, with a diverse range of countries contributing to the scientific literature. The USA stands out as the leading contributor, but other countries from different regions are also actively involved in studying the relationship between acrolein and AD. This collaborative approach fosters a broader understanding of the subject matter and helps in addressing the global challenge of dementia, including AD, as a public health concern.

### Publication of institutions

**FIGURE 5:**
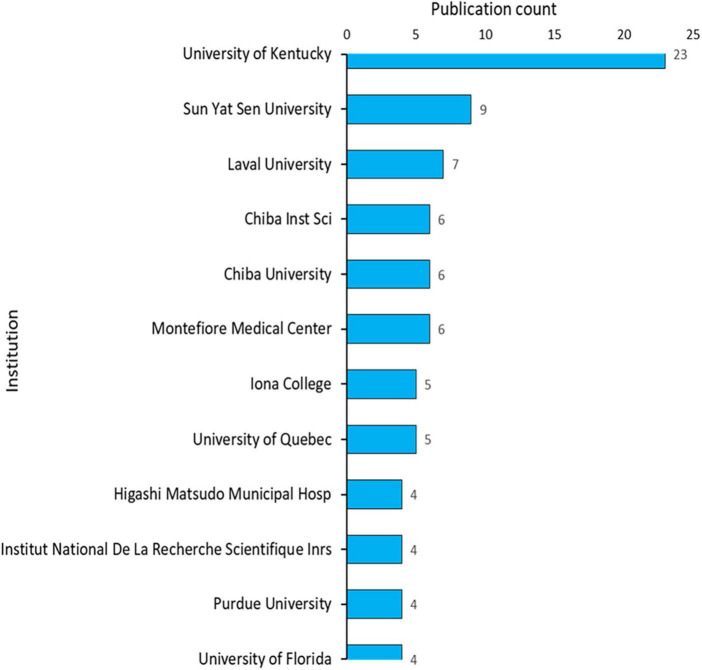
The top 12 institutions with the most publication in acrolein and Alzheimer’s disease research field.

**FIGURE 6:**
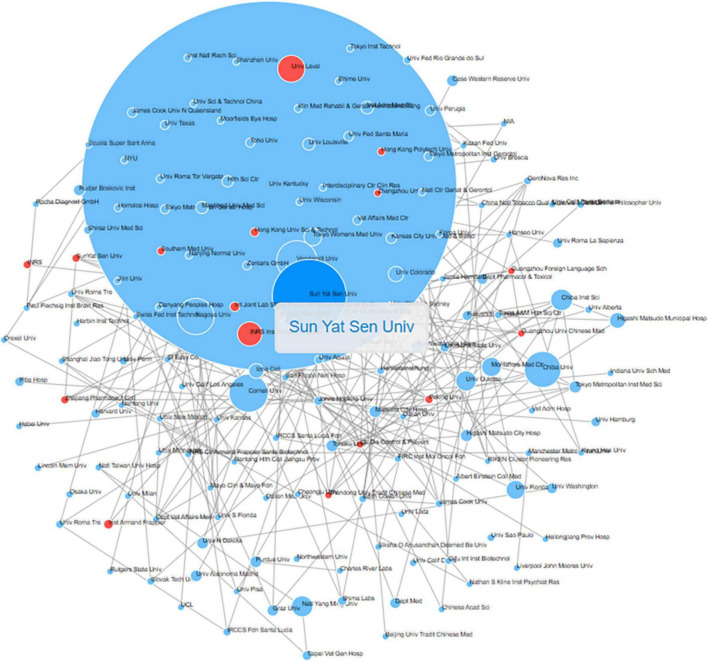
Institutions’ collaboration network map in acrolein and Alzheimer’s disease research field. The blue node represents institution and red node indicates institute collaboration.

The research on acrolein and its association with AD has been a collaborative effort involving a diverse range of institutions. A total of 162 institutions have published articles investigating the relationship between acrolein and AD. Among these institutions, the publication performance of the top 12 contributors is presented in Figure 5. Significantly, the University of Kentucky in the United States has played a prominent role in this research field, contributing 19.2% (*n* = 23) of all the articles on the subject. Following the University of Kentucky, Sun Yat-sen University has contributed 7.5% (*n* = 9) of the articles, and Lava University has contributed 5.8% (*n* = 7). Additionally, we visualized the institution collaborations network map in Figure 6. The institution with the highest collaboration was Sun Yat-sen University, having 16 links, a total link strength of 19.

### Publication of authors

**FIGURE 7:**
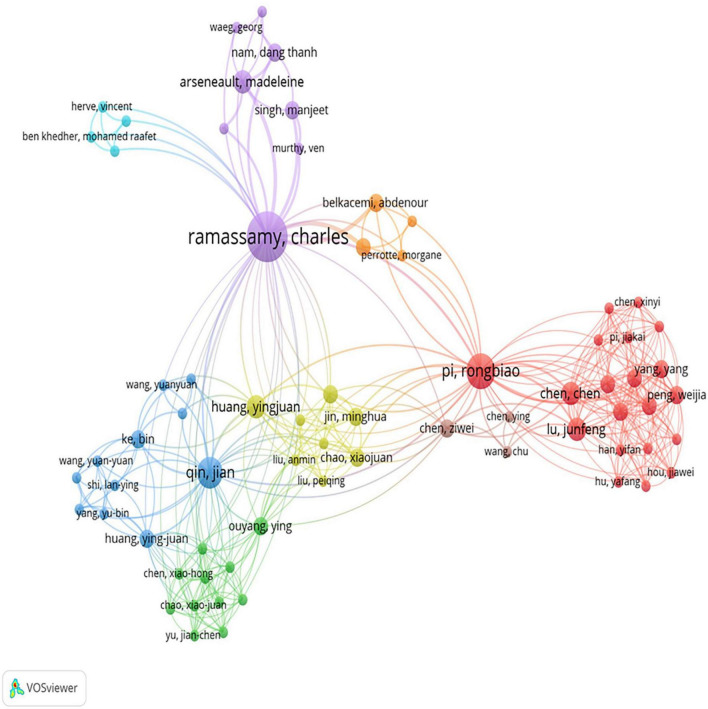
Authors’ collaboration network map in acrolein and Alzheimer’s disease research field. The map consists of 69 author and forms 8 collaboration clusters. Clustering is represented by colors, with authors in the same cluster sharing the same color. The size of circles reflects the frequency of collaboration, with larger circles indicating more collaboration.

A total of 451 authors made contributions to 120 papers related to acrolein and AD, which encompassed information about both the first and corresponding authors in the SCI-Expanded database. The 10 most productive authors in acrolein and AD research are listed in Table 1. Ramassamy C from the institution of Institut National De La Recherche Scientifique (INRS), Canada, ranks first with 11 publications, followed by Butterfield DA and Lovell MA, each with 7 publications. In terms of average citations, Butterfield DA had the most cited documents with 167.7 (Table 1). Figure 7 displays the co-authorship among authors, highlighting the collaboration patterns within the field. It reveals that Pi RB (Pi Rongbiao) from Sun Yat-sen University in China had the most extensive co-authorship collaborations, as indicated by 37 network lines connecting Pi RB to various other authors. This signifies a high level of collaborative activity involving Pi RB. The total link strength, which represents the overall intensity of collaborations, for Pi RB is 55. Following Pi RB, Ramassamy C (Ramassamy, Charles) ranks second in terms of co-authorship collaborations, with a total link strength of 53. This suggests that Ramassamy C is also actively engaged in collaborative research within the field, albeit slightly less extensively than Pi RB. Collaboration could play a crucial role in advancing research in the acrolein and AD domain. The extensive networks built by researchers like Pi RB and Ramassamy C demonstrate the value of collaborative efforts in generating knowledge, sharing expertise, and driving innovation within this research area. Such collaborative activities likely contribute to the development of more comprehensive and impactful solutions for understanding and addressing challenges related to acrolein and AD.

**TABLE 1:** Top 10 authors with most publication.

Rank	Author	Publication	%	Citations	Average citations
1	Ramassamy C	11	9.2	443	40.3
2	Butterfield DA	7	5.8	1174	167.7
3	Lovell MA	7	5.8	977	139.6
4	Igarashi K	6	5.0	144	24.0
5	Kashiwagi K	6	5.0	144	24.0
6	Lopachin RM	6	5.0	543	90.5
7	Pi RB	6	5.0	132	22.0
8	Gavin T	5	4.2	452	90.4
9	Qin J	5	4.2	147	29.4
10	Yoshida M	5	4.2	130	26.0

### Journals and impact factor

The acrolein and AD papers were found within 19 journals. Table 2 presents the top 10 journals along with their respective impact factors. The leading journal in terms of the number of papers published on the subject is the “Journal of AD,” accounting for 11.7% of the total publications. This journal holds a significant impact factor of 4.0, indicating its prominence in the scientific community. Following closely behind is “Neurobiology of Aging” with 5% of the articles and an impressive impact factor of 5.133. The journals “Free Radical Biology” and “Chemical Research in Toxicology” each contribute 3.3% of the articles and have impact factors of 8.101 and 3.973, respectively (Table 2).

**TABLE 2:** Top 10 journals with most publications.

Journal	Publication	%	Citations	Average citations	Impact factor (IF)
Journal of Alzheimer’s Disease	14	11.7	59	4.2	4.00
Neurobiology of Aging	6	5.0	111	18.5	5.133
Free Radical Biology and Medicine	4	3.3	49	12.3	8.101
Chemical Research in Toxicology	4	3.3	21	5.3	3.973
Toxicological Sciences	3	2.5	26	8.7	4.109
Clinica Chimica Acta	3	2.5	20	6.7	6.314
Biochimica et Biophysica Acta-Molecular Basis of Disease	3	2.5	19	6.3	6.633
Toxicology Letters	3	2.5	18	6.0	4.271
Brain Research	3	2.5	11	3.7	3.61
Toxicology and Applied Pharmacology	3	2.5	8	2.7	4.46

### Prolific cited papers

The top 15 most cited acrolein and AD related paper are listed in Table 3. These papers consist of both original articles and reviews documents. All the papers received >100 citations. The total number of citations for the top 15 cited papers was 4,967. The article entitled: “Metals, Oxidative Stress and Neurodegenerative Disorders,” published in the journal of *Molecular and Cellular Biochemistry* with impact factor of 3.842, was the most cited paper with a total citation of 766. Four of the top 15 papers were published in *Neurobiology of Aging* with *IF*_2023_ of 5.133. Out of the 15 papers, three were published by the author “Butterfield, DA” and two by the author “Lovell, MA.”

**TABLE 3:** The top 15 most cited papers in the field.

Rank	Publication title	Total citation	References
1	Metals, oxidative stress and neurodegenerative disorders	766	Jomova et al., 2010
2	Evidence that amyloid beta-peptide-induced lipid peroxidation and its sequelae in Alzheimer’s disease brain contribute to neuronal death	550	Butterfield et al., 2002
3	Acrolein is increased in Alzheimer’s disease brain and is toxic to primary hippocampal cultures	369	Lovell et al., 2001
4	Lipid peroxidation in aging brain and Alzheimer’s disease	368	Montine et al., 2002
5	Oxidative DNA damage in mild cognitive impairment and late-stage Alzheimer’s disease	359	Lovell and Markesbery, 2007
6	Protein-bound acrolein: a novel marker of oxidative stress in Alzheimer’s disease	340	Calingasan et al., 1999
7	Increased levels of 4-hydroxynonenal and acrolein, neurotoxic markers of lipid peroxidation, in the brain in mild cognitive impairment and early Alzheimer’s disease	317	Williams et al., 2006
8	Molecular mechanisms of acrolein toxicity: relevance to human disease	308	Moghe et al., 2015
9	Cytochrome c oxidase and mitochondrial F1F0-ATPase (ATP synthase) activities in platelets and brain from patients with Alzheimer’s disease	288	Bosetti et al., 2002
10	Lipoic acid as an anti-inflammatory and neuroprotective treatment for Alzheimer’s disease	258	Maczurek et al., 2008
11	Involvements of the lipid peroxidation product, HNE, in the pathogenesis and progression of Alzheimer’s disease	213	Butterfield et al., 2010
12	Lipoic acid as a novel treatment for Alzheimer’s disease and related dementias	212	Holmquist et al., 2007
13	Molecular mechanisms of 4-hydroxy-2-nonenal and acrolein toxicity: nucleophilic targets and adduct formation	211	LoPachin et al., 2009
14	Copper and oxidative stress in the pathogenesis of Alzheimer’s disease	209	Eskici and Axelsen, 2012
15	Elevated protein-bound levels of the lipid peroxidation product, 4-hydroxy-2-nonenal, in brain from persons with mild cognitive impairment	199	Butterfield et al., 2006

### Keywords, clusters, and burst words

**FIGURE 8:**
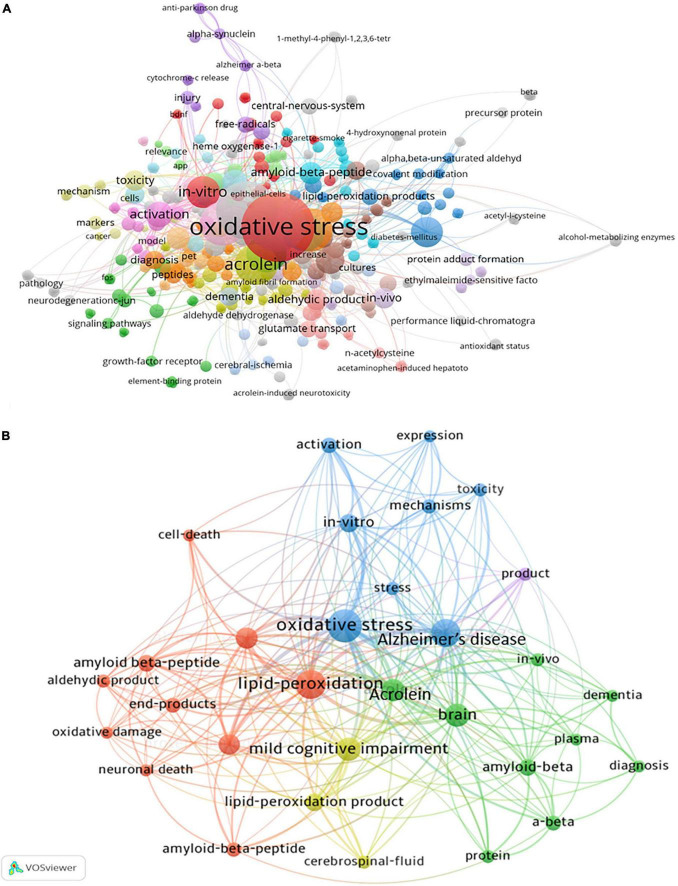
Analysis of keywords in acrolein and Alzheimer’s disease research field. **(A)** Illustrates the complete network of 585 keyword co-occurrence map. **(B)** Shows the top 30 keyword co-occurrence network map. Clustering is represented by colors, with keywords in the same cluster sharing the same color. Circle size reflects keyword occurrence, with larger circles indicating more occurrences.

Analysis of keywords in article could serve as useful tool for identification of research hotspots and frontiers in a certain filed. In the present study, we identified an overall of 585 keywords from the 120 articles in Web of Science Core Collection (Figure 8A). Among these keywords, we subsequently identified the 31 most related keywords linking acrolein and AD (Figure 8B). The most significant keywords linking acrolein and AD were oxidative stress, lipid-peroxidation, acrolein amyloid-beta, alzheimer’s-disease, mild cognitive impairment, mechanisms, activation, cerebrospinal-fluid, end-products, aldehydic product, *in vivo*, and *in vitro* (Table 4).

**TABLE 4:** Top 20 keywords in acrolein and AD research.

Keyword	Occurrences	Total link strength
Oxidative stress	51	132
Lipid-peroxidation	37	120
Alzheimer’s-disease	34	87
Brain	23	75
Mild cognitive impairment	22	73
Acrolein	20	73
Alzheimer’s-disease brain	18	59
Protein-bound acrolein	17	49
*In vitro*	13	37
Lipid-peroxidation product	12	27
Amyloid beta-peptide	10	33
Amyloid-beta	10	32
Activation	9	26
A-beta	8	20
End-products	8	34
Amyloid-beta-peptide	7	16
Mechanisms	7	18
Aldehydic product	6	23
Cerebrospinal-fluid	6	19
*In vivo*	6	15

**FIGURE 9:**
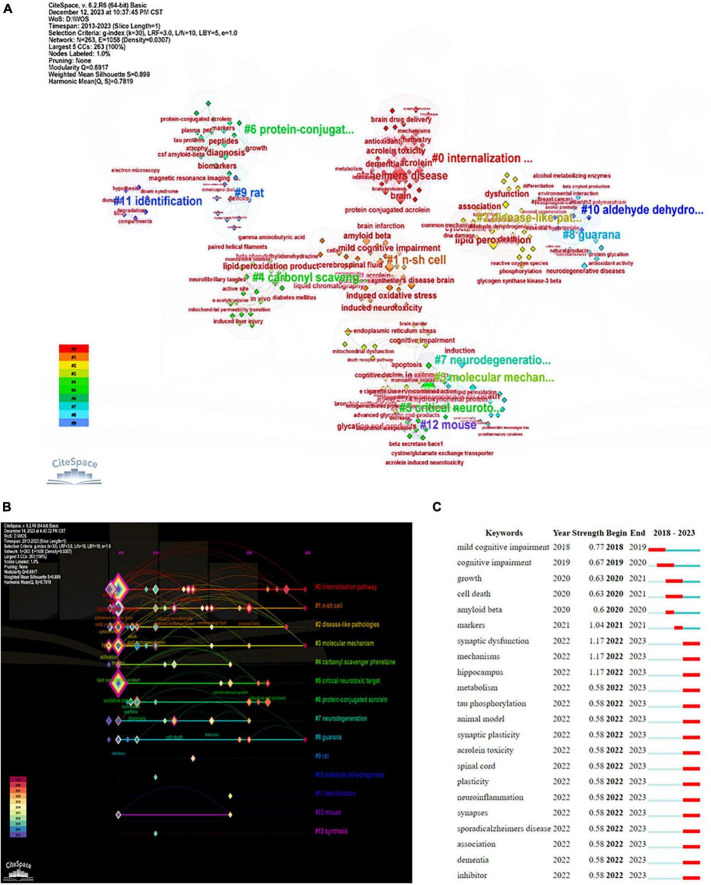
Research hotspots and frontiers in the field. **(A)** Represents the map of the top 15 hotspot clusters. These clusters are categorized into subjects, represented in different colors, with an identification number (ID) ranging from #0 to #15. The smaller the ID number, the greater the cluster, and vice versa. **(B)** Shows the timeline view of co-cited clusters including cluster subjects. This view effectively shows the variations in the emergence time and duration of clusters. The lines and labels transitioning from purple to red indicate more recent research areas. **(C)** Lists the top 22 keywords with the strongest citation burst. The strength value represents the frequency of citations. The red indicator represents the period during which a keyword gains attention.

Using CiteSpace, we identified 15 major hotspot clusters from papers published between 2013 and 2023 (Figure 9A and Table 5). The largest cluster (#0) comprises 42 members and a silhouette value of 0.857. It is labeled as “*internalization pathway*” by log-likelihood ratio and “*assessing acrolein*” (0.87) by mutual information. The most three cited members in this cluster were *Alzheimer’s disease*, *brain*, and *acrolein*. Cluster (#1) is the second largest with 34 members and a silhouette value of 0.76. It is labeled as “*n-sh cell*” by log-likelihood ratio and “*assessing acrolein*” (0.28) by mutual information (Table 5).

**TABLE 5:** The largest 15 clusters in acrolein and AD research.

Cluster ID	Three must cited keywords	Size (SV)	Label (LLR)	Label (MI)	Average year
#0	Alzheimer’s disease, brain, and acrolein	42 (0.857)	Internalization pathway (20.33, 1.0E-4)	Assessing acrolein (0.87)	2017
#1	Mild cognitive impairment, Alzheimer’s disease brain, and amyloid beta	34 (0.76)	N-sh cell (18.75, 1.0E-4)	Assessing acrolein (0.28)	2017
#2	Lipid peroxidation, association, and dysfunction	33 (0.889)	Disease-like pathologies (18.29, 1.0E-4)	Oxidative modification (0.3)	2015
#3	*In vitro*, activation, and A-beta	26 (0.949)	Molecular mechanism (17.65, 1.0E-4)	Acrolein toxicity (0.33)	2018
#4	Lipid peroxidation product, *in vivo*, and beta phenylethylidenehydrazine	22 (0.985)	Carbonyl scavenger phenelzine (8.74, 0.005)	Carbonyl scavenger phenelzine (0.02)	2015
#5	Oxidative stress, cognitive impairment, and glycation end products	19 (0.908)	Critical neurotoxic target (9.5, 0.005)	Assessing acrolein (0.74)	2019
#6	Diagnosis, growth, and peptides	19 (0.908)	Protein-conjugated acrolein (15.18, 1.0E-4)	Polyamine biomarker (0.04)	2016
#7	Cell death, expression, and induction	17 (0.889)	Neurodegeneration (12.16, 0.001)	Joint action (0.11)	2016
#8	Inhibition, apolipoprotein, and neurodegenerative diseases	14 (0.884)	Guarana (12.16, 0.001)	Neurotoxicity (0.11)	2017
#9	Myelin damage, hydralazine, and disease	9 (1)	Rat (8.74, 0.005)	Rat (0.02)	2017
#10	Environmental interaction, aldh2 polymorphism, and alcohol metabolizing enzymes	7 (0.991)	Aldehyde dehydrogenase (8.34, 0.005)	Aldehyde dehydrogenase (0.03)	2015
#11	Hypothesis, beta, compartments	7 (1)	Identification (9.25, 0.005)	Identification (0.02)	2019
#12	Apoptosis, aggregation, and erk	6 (0.992)	Mouse (8.34, 0.005)	Mouse (0.03)	2018
#13	1st radiosynthesis, agents, and cinnamaldehydes	5 (1)	Synthesis (10.97, 0.001)	Synthesis (0.02)	2015
#14	Alpinetin, isomerase, and hesperetin	3 (1)	Acrolein-trapping capacity (8.34, 0.005)	Acrolein-trapping capacity (0.03)	2021

SV, silhouette value; LLR, log-likelihood ratio; MI, mutual information.

CiteSpace software tool generated a timeline maps of the clusters and identify keyword bursts. In this study, the focus of acrolein and AD research has undergone a shift in the past decade. Key areas of interest have transitioned from topics such as “synthesis,” “mouse model,” “identification,” “aldehyde dehydrogenase,” “rat model,” “guarana,” “neurodegeneration,” “protein-conjugation acrolein,” “critical neurotoxic target,” “carbonyl scavenger phenelzine,” “molecular mechanism,” “disease-like pathology,” and “n-sh cells” to the current emphasis on the “internalization pathway” (Figure 9B).

The timeline map that visually represent the evolution of clusters, consisting groups of related keywords or topics, over time is represented in Figure 9B. The study noted a significant shift in the focus of acrolein and AD research over the past decade. Initially, researchers were interested in various topics such as synthesis methods, animal models (like mouse and rat models), identification techniques, specific enzymes (like aldehyde dehydrogenase), natural compounds (like guarana), neurodegeneration processes, protein-conjugation with acrolein, targets for neurotoxicity, treatments (like carbonyl scavenger phenelzine), molecular mechanisms, disease-like pathology studies, and specific cell types (like n-sh cells). However, the current emphasis in research has shifted toward the “internalization pathway.” This indicates a focus on understanding how acrolein enters cells and affects cellular processes, which is crucial in the context of AD.

CiteSpace also identified keyword bursts, marking periods of increased attention or activity around specific keywords, thereby indicating topics of sudden prominence in research. The top 22 keywords with the strongest citation bursts are represented in Figure 9C. In the period between 2021 and 2022, the keywords marker, synaptic dysfunction, mechanism, and hippocampus received notable attention in the field. As illustrated in the figure, the hotspots have evolved, moving from cognitive impairment, cell growth and death, and Aβ deposition to related markers. Subsequently, the focus shifted to synaptic dysfunction, mechanisms, hippocampus, metabolism, tau phosphorylation-related mechanisms, and further to dementia and identifying inhibitors. In addition, 16 keywords exhibited citation bursts from 2022 to 2023.

The analysis of acrolein and AD publications from 1999 to July 2023 reveals a dynamic and collaborative research landscape. The USA emerges as a major contributor, reflecting global efforts in understanding acrolein’s role in AD. Institutions like the University of Kentucky and Sun Yat-sen University, along with prolific authors such as Ramassamy C and Butterfield DA, have significantly advanced knowledge in this area. Leading journals like the “Journal of AD” and “Neurobiology of Aging” have been instrumental in disseminating research findings. Keyword analysis underscores the importance of A-beta, oxidative stress, lipid oxidation, and the emerging focus on the “internalization pathway” in understanding AD pathology related to acrolein exposure. These findings highlight ongoing efforts to address the complex challenges posed by AD. Overall, the study showcases a collaborative and evolving research landscape, with an emphasis on key themes and advancements in acrolein-AD research. This comprehensive analysis contributes valuable insights to the broader understanding of AD and informs future research directions in this critical domain.

## Discussion

Acrolein and AD have been subjects of research due to their potential association and implications in neurodegenerative processes. While the link between acrolein and AD is intriguing, it is important to note that AD is a complex and multifactorial disorder with multiple contributing factors. Acrolein is just one of many potential factors that could play a role in the disease process.

### Characteristics of publication outputs and citation impact

The findings from the analysis of 120 English documents related to acrolein and AD pathogenesis research in SCI-Expanded of Web of Science from 1999 to 2023 suggest significant changes in publication and citation trends in the field. During this period, acrolein and AD research garnered a considerable level of interest, leading to a substantial number of publications in various document types. The 120 English documents encompass a range of studies, including original research articles, reviews, and other specialized types of publications. This demonstrates the depth and breadth of research being conducted in this area.

However, the analysis also indicates a noticeable decline in the rate of new publications in recent years compared to previous years when interest in the topic was more prominent. This decline in publication rate may signal changes in the dynamics of research in the field. Several factors could contribute to this decline. One possible reason could be the saturation of research in the acrolein and AD pathogenesis field. As more studies are conducted and key findings are established, researchers may be focusing on other areas of investigation or moving toward exploring new frontiers in neuroscience and neurodegenerative disorders. Another potential factor influencing the decline in publication rate could be a shift in research focus. Scientific advancements and breakthroughs often redirect attention to different aspects of a research field (Larsen and von Ins, 2010).

As new findings emerge, researchers may choose to investigate different facets of acrolein and AD pathogenesis, leading to a shift in the overall research landscape (Engels et al., 2012; Wahid et al., 2022). Additionally, changes in funding priorities may also contribute to the observed trend. Shifting funding priorities can influence the allocation of resources to specific research areas, potentially leading to fluctuations in the number of new publications over time (Korytkowski and Kulczycki, 2019; Wahid et al., 2022). For researchers and stakeholders in the field, it is essential to take these trends into account when planning future studies and exploring new avenues of investigation. Understanding the current state of research and the factors impacting publication trends can help guide future research directions, inform resource allocation, and facilitate collaborative efforts to address critical gaps in knowledge.

### Global participation: countries, institutions, and authors

This wide representation of countries, institutions and authors in the scientific literature on acrolein and AD highlights the global nature of research efforts in understanding this neurodegenerative condition. The USA notably stands out as the leading contributor, showcasing its strong involvement in advancing the understanding of AD and its potential link to acrolein, especially in the past two decades. However, there has been a decline in their contributions in recent years. The possible reason for this decline in the field could be the saturation of research, which occurs when fundamental questions in the field have been extensively answered, resulting in fewer novel findings and subsequently fewer publications. Additionally, shifts in research focus can occur due to technological advancements, changes in funding priorities, or emerging societal needs, causing researchers to divert their attention from certain topics. Furthermore, as knowledge in a field matures, researchers may focus more on refining existing theories or methodologies rather than making groundbreaking discoveries, leading to a slowdown in publication output. The USA’s active participation and top rank in academic research have been consistently reported in previous studies (Jallow et al., 2020; Wang et al., 2023), reaffirming its significant role in scientific advancements. However, it is essential to acknowledge the active engagement of other countries from different regions, as this collaborative approach fosters a broader understanding of the subject matter and aids in addressing the global challenge of dementia, including AD, as a significant public health concern (Sexton et al., 2021; Alzheimer’s Association, 2023). To foster international research collaboration, a comprehensive approach involves establishing collaborative networks and consortia among researchers, institutions, and funding agencies from different countries (Adams, 2012). These networks facilitate information sharing, joint funding opportunities, and cross-border projects. Promoting joint research initiatives through agreements, grants, and partnerships encourages diverse researchers to collaborate on common goals. Supporting exchange programs enhances collaboration by sharing knowledge and fostering cultural understanding. Improving communication with online tools and open-access platforms promotes global research transparency. Encouraging multidisciplinary projects leverages diverse expertise, while simplifying visas and providing mobility support aids seamless collaboration. Lastly, promoting cultural sensitivity ensures equitable participation and enriches collaborative research (Gewin, 2018; Nature., 2021).

The research on the association between acrolein and AD has indeed been a collaborative effort involving a diverse range of institutions. Notably, the University of Kentucky in the United States and Sun Yat-sen University in China have emerged as prominent contributors in this research field, making significant contributions in terms of publication output, collaboration, and citations. Both the University of Kentucky and Sun Yat-sen University have demonstrated substantial publication output, publishing a considerable number of articles on the subject. Their contributions have been instrumental in advancing the understanding of the relationship between acrolein and AD. Furthermore, these institutions have actively engaged in collaborations with other research entities, fostering a network of cooperative efforts in the field. Collaboration among institutions is crucial for tackling complex scientific questions and sharing expertise and resources. In addition to their publication and collaboration efforts, the research from these institutions has garnered a notable number of citations, indicating the impact and influence of their work on the scientific community. Citations serve as a measure of the relevance and importance of research findings and indicate the recognition of contributions by other researchers in the field. The involvement of institutions from different countries, such as the University of Kentucky in the United States and Sun Yat-sen University in China, highlights the global nature of research on acrolein and AD. This international collaboration fosters a rich exchange of ideas, diverse perspectives, and a broader understanding of the subject matter.

Author participation in acrolein and AD research has been significant, with a total of 451 authors making contributions to 120 papers in the field. This indicates a diverse and collaborative effort involving numerous researchers from different institutions and regions. The top 10 most productive authors in this research area have played a crucial role in advancing the knowledge in acrolein and AD. These authors’ active engagement reflects their dedication and expertise in the subject matter. Additionally, the impact of research output is measured by the number of citations received by the authors’ works. These authors’ documents, received >100 citations, making them highly influential in the field, indicating the significance and recognition of their research findings by other researchers. The collaborative nature of this research is evident from the co-authorship network among authors. Authors like Pi Rongbiao and Ramassamy C are the leaders in co-authorship network, fostering connections and collaborations with various authors around the globe. The active participation of authors from different institutions and countries underscores the global nature of this research effort. Such collaboration allows for the integration of diverse perspectives, expertise, and methodologies, ultimately enriching the overall understanding of the association between acrolein and AD. This collective effort by authors worldwide has contributed to advancements in diagnosis, treatment, and prevention strategies, making a significant impact on addressing AD as a critical public health concern.

### Identifying research hotspots, frontiers, and emerging trend

The analysis of keyword co-occurrences proves highly valuable for pinpointing central themes, hotspots, and emerging areas of significance within a specific field. In this particular study, the VOSviewer analytical tool was employed to investigate both the keywords found within the titles. The objective was to unveil prevalent focal points and dominant domains within this realm of research. The findings show that acrolein continued to attract considerable interest in research on AD pathogenesis. Prominent primary keywords that emerged within this field belongs to acrolein and AD pathology. Notably, these keywords surfaced frequently across studies pertaining to acrolein and its role in the development of AD. The primary focus of these keywords lies in the intricate interplay between acrolein and AD, including its processing, protein interactions, and implications within the brain. It is noteworthy that this analysis highlights a strong connection between acrolein and research related to AD. The prevalence of these keywords indicates substantial interest and ongoing exploration into the possible relationship between oxidative stress, lipid peroxidation, and the role of acrolein in brain health and the progression of AD. The pivotal role of oxidative stress and Aβ in the onset and progression of AD is widely recognized. In recent years, the utilization of antioxidants and anti-amyloid therapy has gained popularity as a prominent strategy in the treatment of AD (Pritam et al., 2022; Zhang et al., 2023).

Through the analysis of keyword clusters, network maps, and keyword bursts, we have successfully identified significant topics and frontiers in the subject domain. In this study, the focus of acrolein and AD research has undergone a shift in the past decade. Indeed, the emergence of the “internalization pathway” as the largest cluster in the field has captured our attention. This suggests a significant focus and potentially pivotal insights within the context of acrolein and AD research. The prominence of this cluster underscores its importance and may indicate a critical aspect of the subject domain that warrants further investigation and exploration. Moreover, the “internalization pathway” cluster appears to encompass the most recent trending hotspots, including synaptic dysfunction, mechanisms, hippocampus, metabolism, tau phosphorylation, synaptic plasticity, acrolein toxicity, association, dementia, and inhibitors (Figure 9C). Indeed, elements such as brain drug delivery, antioxidants, brain chemistry, and infarction are also noteworthy and merit attention. These trends signify dynamic and evolving areas within the research landscape, indicating potential avenues for advancements and breakthroughs in our understanding of AD and acrolein-related mechanisms.

### Internalization pathway

The internalization pathway is a crucial cellular process that involves the uptake of substances from the external environment into the cell. This broad term encompasses various types of endocytosis, each serving distinct purposes in cellular processes (Marsh and McMahon, 1999). The specific internalization pathway employed by a cell depends on the nature of the material being taken up and the mechanisms involved in the process (Schwab, 2001). Receptor-mediated endocytosis (clathrin-mediated endocytosis), caveolae, pinocytosis, and phagocytosis are among the mechanisms through which substances are internalized (Camblor-Perujo and Kononenko, 2022). This process is essential for diverse cellular functions, including nutrient uptake, signal transduction, and the removal of waste products. Understanding the dynamics of the internalization pathway is critical for unraveling cellular processes and has implications for various physiological and pathological conditions.

Amyloid-beta is a peptide that aggregates to form plaques in the brains of individuals with AD (Chen et al., 2017). Aβ aggregation has been shown to implicate multiple pathways in AD, including oxidative stress, inflammatory cascade, and caspase activation, which ultimately lead to neuronal damage (Sehar et al., 2022; Iliyasu et al., 2023). Mounting evidence indicates that the processing of APP is influenced by endocytosis and intracellular sorting (Rajendran et al., 2006; Chaufty et al., 2012). Given that the amyloidogenic processing involving β-secretase/γ-secretase occurs within endosomes, the generation of Aβ is reliant on the endocytosis of APP from the cell surface and its subsequent transit to the endosomes. Notably, cells expressing APP with impaired endocytosis capabilities demonstrate a marked reduction in Aβ production (Wu and Yao, 2009).

On the other hand, acrolein is a highly reactive aldehyde and a known byproduct of lipid peroxidation, a process associated with oxidative stress (Chen et al., 2017; Zhu et al., 2022). In AD, elevated levels of acrolein have been detected in the brains of affected individuals (Sanotra et al., 2023). While acrolein is known to induce cellular damage and exacerbate neuroinflammation, the specific mechanisms through which it enters neuronal cells, and whether its internalization is a significant contributor to AD pathology, are aspects that necessitate further exploration. The intricate interplay between acrolein and the internal cellular processes, particularly endocytosis, in the progression of AD is an area that demands deeper investigation.

### Acrolein and Aβ interaction

**FIGURE 10:**
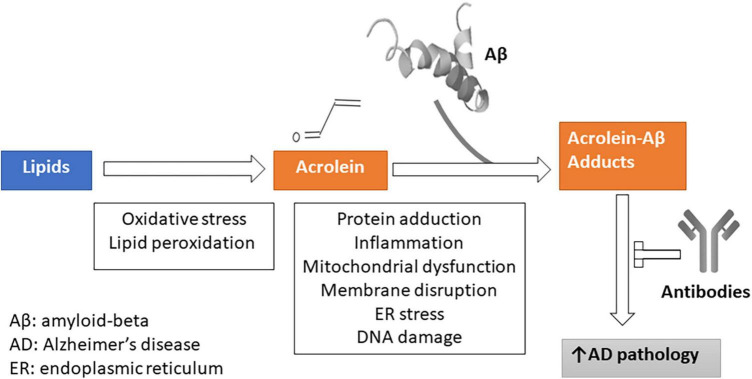
A hypothetical diagram on the complex relationships between acrolein, amyloid-beta, and cellular responses in Alzheimer’s disease pathogenesis. Reactive oxygen species (ROS) from oxidative stress and lipid peroxidation alters lipid metabolic pathways, producing acrolein. Acrolein binds with amyloid beta, forming the harmful acrolein-Aβ adduct. This worsens AD pathology and hinders immune responses. These processes also trigger protein adduction, inflammation, mitochondrial issues, and DNA damage.

Taking advantage of the findings from previous studies in our lab (Sanotra et al., 2023) and the current study, we made a hypothetical diagram to visually demonstrate the complex relationships between acrolein, Aβ, and cellular responses in AD pathogenesis (Figure 10). The alteration of lipid metabolic pathways, targeted by oxidants such as ROS following oxidative stress, leads to the formation of lipid peroxidation through polyunsaturated fatty acids. This process subsequently results in the production of acrolein, an end-product of lipid peroxidation. However, the increased production of acrolein initiates the recruitment of amyloid beta, ultimately resulting in the formation of the acrolein-Aβ adduct. The heightened presence of this adduct exacerbates the pathology of AD and hampers the natural immune response mediated by antibodies against AD onset. On the other hand, these modified metabolic processes trigger a cascade of responses, including protein adduction, inflammatory reactions, mitochondrial dysfunction, membrane disruption, endoplasmic reticulum stress, and DNA damage.

### Strengths and limitations

To the best of our knowledge, our study marks the first bibliometric analysis conducted in the realm of acrolein and AD pathogenesis. This study has effectively unearthed the historical perspective of publications and citations within this field, identified major contributors including countries, institutions, and authors, and illuminated research hotspots and frontiers in AD research. The data employed for our study was meticulously extracted solely from the WoS, thereby ensuring the utilization of a comprehensive, reliable, and widely acknowledged dataset. It is pertinent to highlight that previous bibliometric analyses have similarly drawn from this database for their research (Jallow et al., 2021; Fei et al., 2022; Sun et al., 2022), further affirming its credibility and extensive adoption within the scientific community.

Nevertheless, this study does carry certain limitations. Firstly, due to the recurrence of the same author abbreviations in articles and the inability of bibliometric software to differentiate the contributions of authors with identical names, some degree of accuracy loss may remain unavoidable despite our efforts to rectify this issue. Secondly, while we have endeavored to organize the principal research contributions from the primary literature, the analysis remains a work in progress, and researchers are encouraged to delve deeper into the literature to uncover additional meaningful research trajectories. Thirdly, relying solely on WoS for article searches in bibliometric analysis may introduce biases by favoring high-impact and established journals, potentially overlooking valuable contributions from newer or niche journals. Additionally, WoS’s incomplete data coverage can lead to gaps in the analysis, missing significant contributions. Furthermore, the time lag in indexing within WoS may affect the accuracy and timeliness of bibliometric analyses, particularly in rapidly evolving research areas.

## Conclusion

Overall, research on acrolein and its potential role in AD involves collaboration between countries, institutions, and authors, reflecting the global effort to tackle the challenges posed by dementia and neurodegenerative conditions. The Journal of Alzheimer’s Disease has garnered the highest number of publications in this field, solidifying its preeminent status. The United States emerges as the most prolific country concerning publications, citations, and international partnerships within this domain. The University of Kentucky in the United States takes the lead as the most productive institution, while Sun Yat-sen University boasts the highest count of international collaborations. Author “Ramassamy C” has notably published a majority of the papers within this field. The co-occurrence of keywords, keyword clusters, and burst detection has illuminated the cross-talk between acrolein and AD. The major focus in the field includes oxidative stress, lipid peroxidation, Aβ, and cognitive impairment. An emerging trend in the research category appears to be the involvement of the internalization pathway, extending to areas such as synaptic dysfunction, mechanisms, hippocampus, metabolism, tau phosphorylation, synaptic plasticity, acrolein toxicity, neuroinflammation, association, dementia, and inhibitors. It is important to underscore that research into acrolein and AD remains ongoing, necessitating further studies to comprehensively unravel the depth of their correlation and potential implications in the onset and progression of AD. As our knowledge advances, this area of research may open new avenues for therapeutic interventions or preventive strategies for AD.

## Data availability statement

The original contributions presented in this study are included in the article/supplementary material, further inquiries can be directed to the corresponding author.

## Author contributions

AJ: Conceptualization, Data curation, Formal analysis, Investigation, Methodology, Software, Validation, Visualization, Writing – original draft. DN: Data curation, Investigation, Writing – original draft. MS: Data curation, Investigation, Writing – review & editing. C-HH: Funding acquisition, Resources, Writing – review & editing. Yi-FL: Project administration, Resources, Writing – review & editing. Yu-FL: Conceptualization, Funding acquisition, Project administration, Resources, Supervision, Validation, Writing – review & editing.
